# *ARENA*—Augmented Reality to Enhanced Experimentation in Smart Warehouses

**DOI:** 10.3390/s19194308

**Published:** 2019-10-04

**Authors:** Luis Piardi, Vivian Cremer Kalempa, Marcelo Limeira, André Schneider de Oliveira, Paulo Leitão

**Affiliations:** 1 Research Centre in Digitalization and Intelligent Robotics (CeDRI), Instituto Politécnico de Bragança (IPB), Campus de Santa Apolónia, 5300-253 Bragança, Portugal; pleitao@ipb.pt; 2 Graduate School of Electrical Engineering and Computer Science (CPGEI), Universidade Tecnológica Federal do Paraná (UTFPR), Avenida 7 de Setembro 3165, 80230-901 Curitiba, Paraná, Brazil; vivian.kalempa@udesc.br (V.C.K.); limeira@alunos.utfpr.edu.br (M.L.); andreoliveira@utfpr.edu.br (A.S.d.O.); 3 Centro de Educação do Planalto Norte (CEPLAN), Universidade do Estado de Santa Catarina (UDESC), Rua Luiz Fernando Hastreiter 180, 89283-081 São Bento do Sul, Santa Catarina, Brazil

**Keywords:** smart factories, augmented reality, multi-robot, virtual LRF

## Abstract

The current industrial scenario demands advances that depend on expensive and sophisticated solutions. Augmented Reality (AR) can complement, with virtual elements, the real world. Faced with this features, an AR experience can meet the demand for prototype testing and new solutions, predicting problems and failures that may only exist in real situations. This work presents an environment for experimentation of advanced behaviors in smart factories, allowing experimentation with multi-robot systems (MRS), interconnected, cooperative, and interacting with virtual elements. The concept of ARENA introduces a novel approach to realistic and immersive experimentation in industrial environments, aiming to evaluate new technologies aligned with the Industry 4.0. The proposed method consists of a small-scale warehouse, inspired in a real scenario characterized in this paper, managing by a group of autonomous forklifts, fully interconnected, which are embodied by a swarm of tiny robots developed and prepared to operate in the small scale scenario. The AR is employed to enhance the capabilities of swarm robots, allowing box handling and virtual forklifts. Virtual laser range finders (LRF) are specially designed as segmentation of a global RGB-D camera, to improve robot perception, allowing obstacle avoidance and environment mapping. This infrastructure enables the evaluation of new strategies to improve manufacturing productivity, without compromising the production by automation faults.

## 1. Introduction

The advanced manufacturing introduces intelligent behaviors to improve productivity, allowing more flexible products, in smart factories. This modernization is called the fourth revolution, a technological initiative that started, in Germany, around 2013. This initiative has as a main goal the digitization of processes, transforming traditional factories into smart factories [1]. According to Kagermann et al. [1], Industry 4.0 can be seen as a combination of Information and Communication Technologies (ICT) and Cyber-Physical Systems (CPS) in the manufacturing environment, to optimize the production process through the interaction of interconnected dynamic agents. In this context, smart factories should be composed by a group of agents (i.e., robots and machines) with machine-to-machine connectivity that can interchange information, aiming to take decisions without compromising production and ensuring continuous manufacturing [2].

Industrial environments are evolving due to new emerging technologies, increasing automation level and the use of robotic devices. Mobile robots can adapt to manufacturing changes, addressing the diverse needs of the industry, tracking robotics progress, and incorporating new techniques [3,4]. In smart warehouses, the MRS is deployed to transports goods efficiently, optimizing time and human resources, as happens at Amazon [5], Ocado [6], and DHL [7] warehouses. The automated warehouse with mobile robots has been an important research topic. The Kiva [8] system was one of the first systems to use MRS for warehouse tasks.

A system is considered MRS when there are two or more robots that act in a coordinated (or synchronously) or cooperative (with mutual interaction) way, in the same environment, with the same objectives. The class of MRS is also derived in accordance with the number of agents, i.e., in a low scale it is labeled as multi-robots [9,10], in a medium-scale as a swarm [11] and in a large scale as massively multi-robot [12]. MRS is represented by a group of autonomous robots endowed with artificial intelligence, with the capacity of self-organization, and that cooperates among themselves locally to execute a common goal of the group [13]. The ability to interact locally with other robots in the group provides this robotic system with an individual behavioral characteristic that has the potential for many practical applications. MRS has attractive attributes for industrial processes, specifically for smart warehouses, such as fault tolerance, scalability, and flexibility [14,15].

The MRS brings the collectivity of autonomous agents to industrial scenarios, allowing parallel multi-task and the redundancy to tolerate faults. Several inspirations are adopted to specify collective behaviors, such as nature, achieving swarm intelligence, where simple individual rules can produce a broad set of complex swarming behaviors. Swarm Robotics (SR) involves constant cooperation among individuals who directly reflect on the action of the whole group. The vast majority of the works carried out in this area focus on the study of science related to the collective behavior of swarms [16,17,18]. In this way, the robots are based on characteristics that bring them closer to the features found in nature [19], such as decentralization [20,21] and limited communication between robots [22]. These inspirations are generally conflicting with industrial environments, where the focus is not collective behavior, but the production efficiency.

All these concepts are intended to alter the manufacturing dramatically, turning them into real smart factories. However, scientific and technological development is hampered by the fact that no factories are available for actual experimentation as well as because few industries are interconnected and with a considerable number of mobile agents. Thus, the experimentation with MRS in industrial scenarios is limited to virtual platforms [23] or restricted scenarios [24]. The inability to accurately evaluate the unmodeled disturbances (such as lighting, friction, inertia, communication, etc.) can result in discrepancies between validations in real and virtual models.

Despite all the enthusiasm on the MRS theme, this system is currently limited in terms of real applications. According to Salvaro [25], the fact that the system is complex in terms of design and behavior, it is confined to simulated and practical laboratory applications. In addition, Dorigo et al. [26] related that mathematical and statistical models that describe MRS behavior are under development, and a theoretical methodology that represents the dynamic interactions of MRS behaviors is lacking. Thus, the evaluation of MRS is complicated, but essential, and cannot be described without simplifying global behavior.

One solution is to use the AR experience for the representation of industrial environments with MRS since AR provides a methodology for testing techniques and tools that can be reproduced in reality, more flexible than only simulation environment. It allows developers to design a variety of scenarios by introducing any virtual objects in real-world experiments [27,28]. AR is one of the most promising technologies in the context of Industry 4.0 [29] and contributes to industries that want to optimize their systems, avoiding complex simulation models, expensive hardware setup and a highly controlled environment, in the various stages of development of smart factories [27].

In this sense, this work discusses a novel approach to the experimentation of MRS in industrial environments. The new concept of ARENA is introduced and refers to the small-scale representation of a warehouse logistics, managed by an MRS, to the evaluation of new technologies to smart factories. The AR is employed to achieve more immersive and realistic experimentation, allowing the assessment of intelligent behaviors.

This paper is organized as follows. Section 2 presents warehouse logistics and its concepts. Section 3 details the proposed concept to evaluation of smart factories, the ARENA, and discusses all components individually. Experiments and analyses are presented in Section 4. Conclusions and potential future work are drawn in Section 5.

## 2. Warehouse Logistics

**Figure 1:**
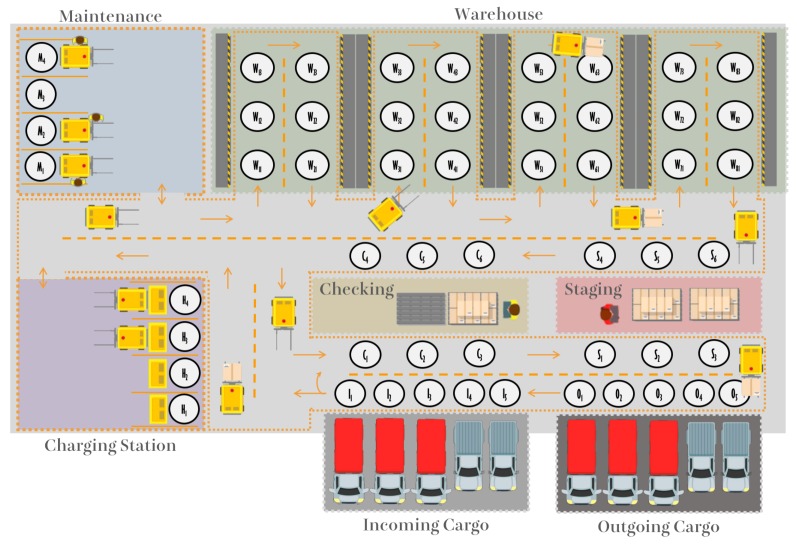
Organization of warehouse logistics.

The inspiration for this work is a real warehouse logistics, located in Brazil, which requires a full automation process. In this way, all sectors such as maintenance, reloading of robots, loading and unloading of products, warehouses, and sorting, among others, were faithfully reproduced in the model. Forklifts can perform different actions inside the represented warehouse, for example, loading and unloading trucks, going to charging station or maintenance area and storing and removing loads from racks. Figure 1 illustrates the floor design of the warehouse, which organizes its process into seven sectors: Incoming Cargo, Outgoing Cargo, Checking, Staging, Warehouse, Charging Station, and Maintenance.

Incoming Cargo is the sector where trucks park so that forklifts can unload the goods. Then, these goods, or packages, are sent to the Checking area. In this sector, the integrity of the received packet is verified, and then a classification is made to determine in which location of the warehouse rack that package will be stored. The warehouse has four aisles, each for one type of goods. The first one is for automotive items, the second for pharmaceutical items, the third for food items, and the last for miscellaneous items. Each aisle has two cabinets, with three shelves each.

**Figure 2:**
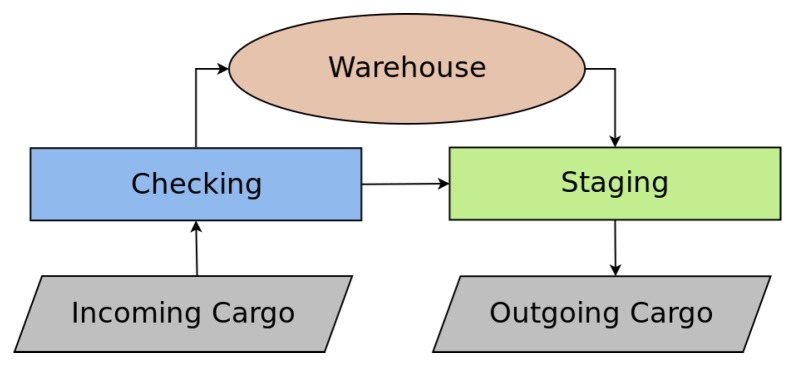
Summary of warehouse logistics process.

Outgoing Cargo is the sector where trucks are loaded with packages to be delivered. Goods must first be removed from the warehouse and then made available in the Staging sector, where they are packed before arriving in the outgoing area. After this process, a forklift loads these packages from the Stating area to the Outgoing Cargo sector. In both the incoming and outgoing goods, if there are many packages to be handled, more than one forklift can be chosen to fulfill the request. The summary of warehouse logistic process is shown in Figure 2.

Two sectors are focused on forklifts support, Maintenance and Charging Station. If the forklift needs to be recharged, it should be directed to the Charging Station sector. Otherwise, if the forklift requires any predictive maintenance or if it breaks, it will need to go to the Maintenance sector.

**Figure 3:**
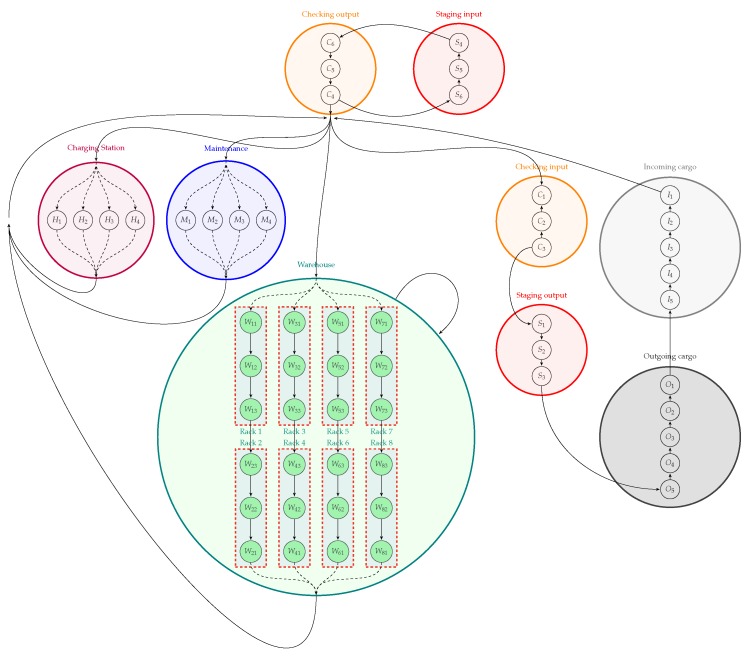
State machine of warehouse logistic process.

The process of warehouse logistics is decomposed into several specific states, and grouped into sector super stages. These stages can be organized in a global state machine that represents the whole warehouse logistics process, as shown in Figure 3. The transitions of the state machine indicate the direction the forklifts can travel, which avoids possible collisions between them. Besides, each state, identified as a white circle in Figure 1 or as a specific state in Figure 3, indicates a position that can be occupied by only one forklift.

Warehouse logistics is a dynamic process with several concurrent tasks due to the entrance and exit of goods co-occurs. Urgent requests must be executed immediately, with time requirements of consumer or storage, e.g., refrigerated cargo. The management is dynamic because the tasks constantly change the number of operators that perform it, to the point that the task can be interrupted by the momentary lack of operators. Several random situations also can change the logistics execution, as a forklift can break or runs out of the battery to complete a task.

## 3. The Concept of the ARENA

**Figure 4:**
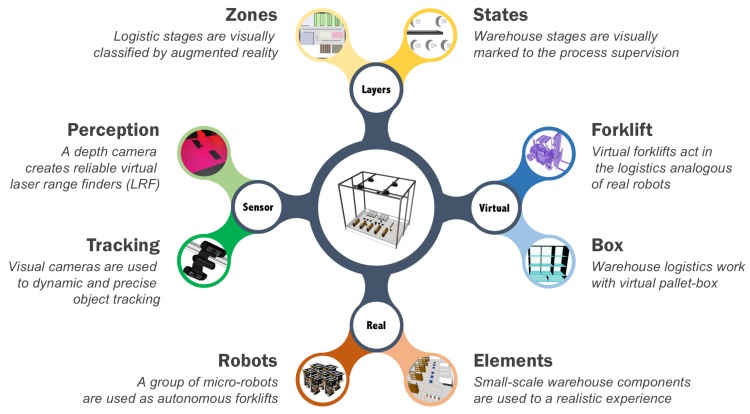
Overview of the ARENA.

The ARENA is a novel concept for active experimentation in smart warehouses, aiming to promote the real characteristics of the factory floor. The approach is a composition of new technologies (as shown in Figure 4), specially designed to evaluate new methods with requirements of Industry 4.0, such as advanced robotics, Internet of things, cloud computing, artificial intelligence, AR, and machine-to-machine.

The project ARENA is organized into four fundamental axes. The *Real* axis is the warehouse infrastructure, where the physical components (trucks, pallets, and racks) are built in small-scale. A group of tiny swarm robots is designed to execute the box handling, with the same mobility of real forklifts.

The AR introduces novel possibilities with the addition of virtual elements, in *Virtual* axis. Thus, tiny robots can handle great boxes, executing the incoming and outgoing cargo actions. New virtual agents can be added on the system to an evaluation in various scenarios, with different kinds of virtual forklifts, or to the analysis of the human–robot interaction with virtual operators.

The *Sensor* axis is related to the global and local perception capabilities. The tiny swarm robots are located inside the warehouse through fiduciary markers, which are detected by a global RGB-D sensor. This camera also provides a global point cloud that is segmented in individual LRF to equip the tiny robots and virtual forklifts.

The immersive experimentation is achieved with the introduction of AR elements to delimit the warehouse zones in the real scenario, in *Layers* axis, allowing the practical analysis of smart managing of cargo. The warehouse logistics is structured in a set of discrete sequential stages, resulting in a warehouse machine state, as shown in Figure 3, and these stages are virtually stamped in the real scenario.

### 3.1. Warehouse Infrastructure

**Figure 5:**
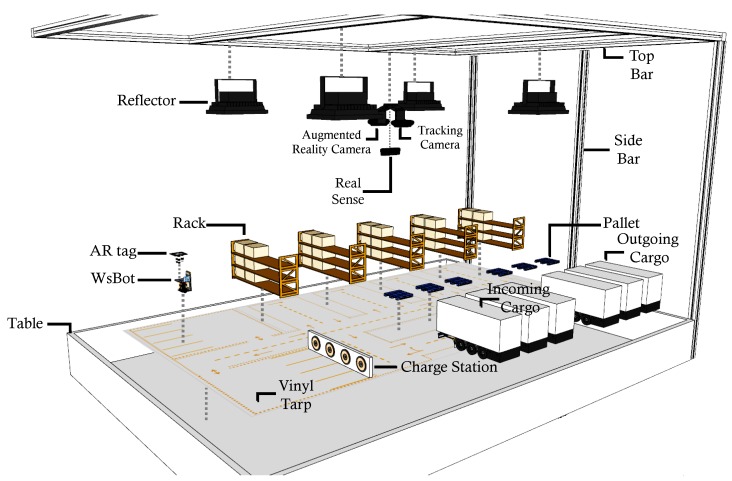
Exploded view of the ARENA’s infrastructure.

The ARENA contains a physical structure that represents the actual dimensions of the warehouse on a smaller scale, providing the same characteristics and operating regions as the real warehouse. Since the inspiration warehouse is not autonomous and is dependent on human intervention or control, the objective is to use the ARENA along with a MRS and AR, to develop an autonomous warehouse system. In this sense, the developed intelligent warehouse system will be validated in the ARENA, and then will be deployed to the real warehouse. The infrastructure gathers the necessary apparatuses to represent the functionalities of the warehouse, as can be seen in Figure 5. The elements that compose it can be classified into static or dynamic components, which are described below.

The infrastructure is mounted over a large custom table that supports the static or dynamic components, with the dimensions of 1.4 m long and 2.1 m wide. This support is composed of wood, and it is located on a vinyl tarp, which is plotted with the industry plant used as a model, i.e., the warehouse. On the side of the table, there is a metal structure which has a height of 1.6 m, used to support cameras and lamps.

Two RGB cameras and one RGB-D camera are employed in the ARENA to represent all the functionality required by the warehouse system developed. An RGB camera is dedicated to tracking robots through image processing by locating the position and orientation of the AR-tags fixed on top of tiny mobile robots, the WsBots. Another RGB camera is used for AR, where virtual layers are added to the original image to achieve a more immersive and realistic experimentation experience. The RGB-D camera introduces the virtual sensors in WsBots, to make the same capabilities of LRF used in real forklifts.

Another important static component present in the ARENA is the charging station. It is made up of four wireless charge transmitters, which have an electronic circuit and a coil to transmit the power to one receiving coil coupled to the robot. Thus, it is possible to autonomously charge the robots without the need to manually connect them to the power or to turn off/stop the robot system.

Real warehouse elements are added to obtain the real environment perception, where are introduce racks, made of wood and designed by a CNC, 3D printed pallets and cardboard boxes. The incoming and outgoing cargo zone is also specified by plastic cargo trucks. All these real elements are designed in scale to real warehouse components.

The forklift actions are executed by the WsBot which interacts with the other features present representing a real warehouse forklift. However, due to its payload limitations, cargo handling is performed in virtual boxes.

### 3.2. The Tiny, Low-Cost Swarm Robot: WsBot

**Figure 6:**
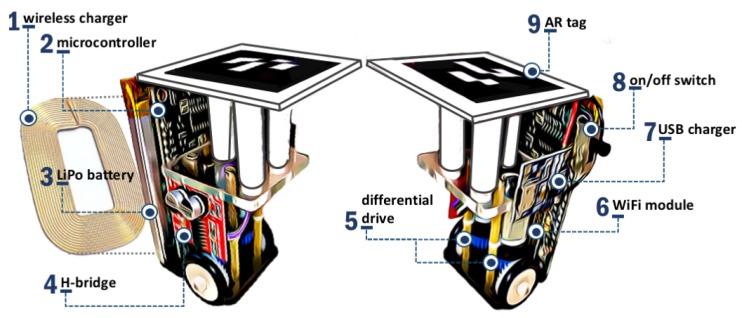
Main components of WsBot.

The WsBot is a tiny robot developed for experimentation in small-scale real warehouses, aiming to evaluate intelligent collective behaviors in the Industry 4.0. The robot is compact, low cost, quick to assemble, with low complexity, and easy to program, as shown in Figure 6.

The robot’s actuation is based in real forklifts with the differential drive system. The prototype has two wheels coupled to independently micro DC motors through the 90-degree gears. The common axis of these two wheels is located just below the center of the base. In this traction system, each engine is connected to a respective side wheel employing a gear assembly. The management of linear and angular velocities is performed by controlling the current supplied to each motor and, consequently, its speed. In this way, the angular velocity will be proportional to the difference between the powers provided for each engine. In place of the castor wheel, a 3 mm LED was used for support, which is adopted due to its small size, resistance, and low coefficient of friction. The mechanical characteristics of WsBot are summarized in Table 1.

The mobile robot has three degrees of freedom ${\left[x,y,\theta \right]}^{T}$ that are controlled by visual feedback, through the visual reference of AR-tag in relation of global RGB camera. The camera identifies all WsBot in the warehouse by AR-tag id and estimates the global position and orientation.

The WsBot is designed for wireless recharging with inductive power transfer (IPT) technology, which allows recharging without any physical connection, only needing to position the robot near to the charging station. The recharging system consists of two circuits: primary circuit and secondary circuit. The primary circuit is the charging station, and it generates a stream of high-frequency current that produces an alternating magnetic field. This field is coupled to the receiver coil on the secondary circuit and induces voltage distribution by Faraday’s law. The secondary circuit then converts this alternating current to continuous and feeds the battery charger driver.

**Figure 7:**
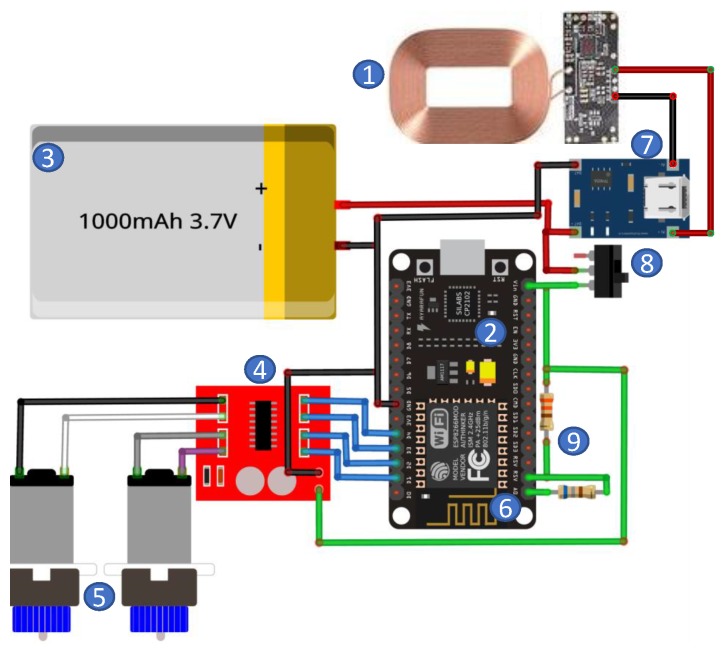
Electronic architecture of WsBot.

The robot’s hardware was especially projected to be quickly assembled and repaired and to simplify the mass production of swarms. The electronic architecture is composed of nine subsystems, as detailed in Figure 7, which are explained individually below.

1. Wireless charger is a copper coil and a circuit board with a voltage regulator and power of 10 W.
2. Microcontroller is responsible for manage motors, acting the H-bridge, and communicate to publish data (in this case battery information) in ROS server. It is embedded in a development board and has 4 MB of flash memory. The microcontroller also has some I/Os able to connect sensors.
3. Lithium Polymer (LiPo battery) is a 3.7 V and 1100 mAh rechargeable battery, with size of 35 mm × 50 mm × 2 mm and 25 g of weight.
4. H-bridge is the dual-driver power circuit to controls motors independently with a current of up to 1.5 A for each engine.
5. Differential drive is a set of small size DC motors that convert electrical impulses in mechanical movement, i.e., robot actuating.
6. WiFi module is an ESP8266 microchip for wireless communications, which allows communication between robots.
7. USB charger is a battery charger capable of recharging without the need to remove it. It is powered by a mini-USB with a voltage of 5 V, providing a voltage of 4.2 V for the battery, with a capacity of 1.2 A.
8. On/off switch is a mechanical switch, with the function of turning the robot on and off. For safety reasons, it is advisable to charge the robot with it turned off.
9. Voltage divider is the resistive circuit, with a coupling two resistors of 330 and 680 Ω respectively, to adjust voltage scale to A/D converter that measure battery state.

### 3.3. Augmented Reality

**Figure 8:**
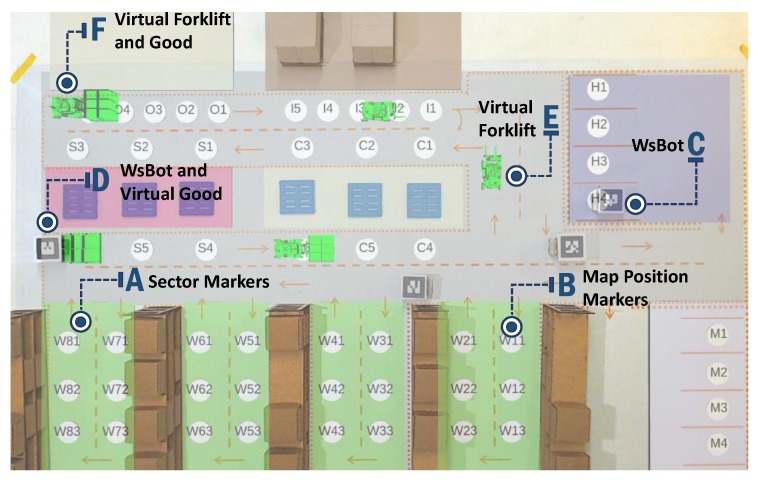
Top view of the AR elements in the ARENA.

The ARENA has virtual elements to improve its experimentation capabilities through the AR, consisting of superimposing virtual graphics on the real workbench. These elements complement warehouse function, e.g., the WsBot can transport goods from truck to rack. The proposed approach combines virtual elements with real components, providing real-time interaction, making their functionality even more complete, and can insert different types of graphic objects. Figure 8 exemplifies the use of AR in smart warehouses experimentation in the ARENA.

Static objects are loaded at system start-up and have their positions already defined in the system. The map position marker is used to determine useful positions within the warehouse (e.g., positioning for loading robots or placing/removing goods from racks), whereas sector markers are used to highlight different sectors within the warehouse (“A” and “B”, respectively). These markers have an essential function for visualization of different environments and occupied positions, and can assist in the development of intelligent warehouse systems.

All dynamic components have positioning changes as they perform certain activities or interact with real objects. The item “C” indicates a WsBot, which, although not a virtual element, integrates AR by the fact of its interaction, carrying virtual goods from one sector to another, as shown in “D”. WsBots also interact with the virtual forklift “E” so that both cannot occupy the same position at the same time. Finally, at “F” in Figure 8, the forklift transports virtual goods across different sectors of the warehouse.

The augmented reality can be employed to verify different situations in the ARENA and to develop new approaches to problems that are currently challenges in a smart warehouse. For example, obstacle avoidance in path planning techniques can be developed and validated using virtual robots interacting with real obstacles or even inserting random failures into real robots such as depleted batteries and enabling virtual robots with systems to recover from these failures.

This proposed hybrid system is capable of interacting the real elements with the virtual ones, with the benefit of reducing the intrinsic inaccuracy of the purely simulated environments. Virtual-developed systems are rarely directly applied in real systems because they are inaccurate models causing a lack of high fidelity replication of real-world, called reality-gap. The ARENA employs the augmented reality and ROS framework to bridge the gap between simulation and reality, proposing a more dynamic testing environment, respecting real-world policies and laws. This approach adopts the advantages of speed, safety, and low cost of simulation, using virtual components, besides having the fidelity of the real models through static and dynamic objects.

### 3.4. Virtual Perception

Most of the industrial mobile robots base their perception of the environment on LRF. These sensors are well accepted due to their high resolution at long distances, with few illumination influences, thus ensuring accuracy and robustness in their measurements. However, WsBot are tiny robots that do not allow the coupling of LRF, due to its reduced size and payload. Thus, it is proposed the development of multiple virtual LRF sensors, for each WsBot, from a single point cloud obtained from a global RGB-D camera, makes it possible to mapping and navigates in the warehouse. In other words, with the data collected from a single RGB-D camera located at the top of the ARENA, it will be possible to embed on each robot an LRF sensor.

**Figure 9:**
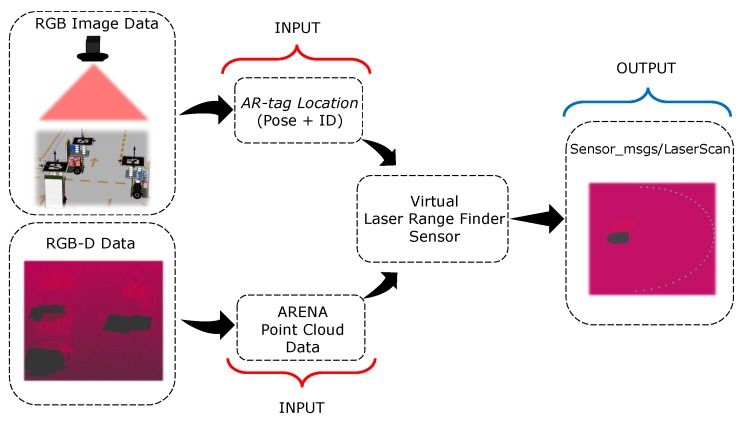
Proposed approach to virtual perception.

The conception of virtual LRF sensor (Figure 9) is made initially by image acquisition of the RGB camera, responsible for tracking the WsBot. In this image, tags attached to the robot structure are arranged in different positions of the ARENA, depending on the activity or state of each robot. The tag ID, position (x,y,z) and quaternion orientation in a frame relative to the RGB camera (*rgb_frame*) is obtained. From these data the identification (ID) of the robot (same as the tag) and also the pose ${\left[x_{i},y_{i},\theta_{i}\right]}^{T}$ from each robot (relative to *rgb_frame*), where i=[0,…,N] represents the index of the *N* robots, are extracted.

The RGB-D camera acquires the depth images from ARENA, through top viewpoint, with a distance of 1.5 m until table center. Each depth image obtained is a group of points (X,Y,Z) of $R^{3}$, relative to (*depth_frame*), and organized in a point cloud. The RGB-D camera is in the center point on the board at the height of 1.5 m from the table base. This distance is considerably higher than any component present in the ARENA, and, for this reason, the influence of hidden areas on the point cloud does not significantly affect virtual sensor behavior. The most significant element and therefore, the one with the most considerable shadow are the racks. They are positioned in centralized regions about point cloud sensor capture, which significantly softens the existence of blind spots because the projection angle is close to zero. Due to the small size of the robot, the shadow it produces is in a blind zone located near the center of the sensor, which is also present in real LRF sensors.

Next, a coordinate transformation from both RGB (*rgb_frame*) and RGB-D (*depth_frame*) cameras to the *world_frame* is performed, the latter being the standard frame of reference for the ARENA. Thus, it is possible to obtain the pose of the robots concerning the world frame, as well as the point cloud data X,Y,Z about the *world_frame*. To simplify the explanation of this section, whenever referring to any position the *world_frame* is used, unless otherwise specified.

Virtual LRF sensors must follow the WsBots motion around ARENA. Thus, LRFs are static in the relation of WsBots but mobile about ARENA. The virtual sensors are attached in the center of AR-tag of WsBots. In other words, the $virtual\_sensor_{i}$ of the $robot_{i}$ will be at the position ${\left[x_{i},y_{i},\theta_{i}\right]}^{T}$ with the height $z_{i}$. Thus, the virtual sensor will perform movements together with the robot, but concerning the robot remains fixed.

The virtual sensors are designed to be identical to real LRF, acquiring information about obstacles around robots. This information is published by the virtual sensor as *sensor_msgs/LaserScan*, following the same pattern used by real LRF sensors, having a defined and standardized structure with time stamp, start angle, end angle, angle increment (number of degrees separating consecutive beams), minimum range, maximum range, and vector of ranges, which stores in order from start to end laser, the distances obtained by the sensor.

**Figure 10:**
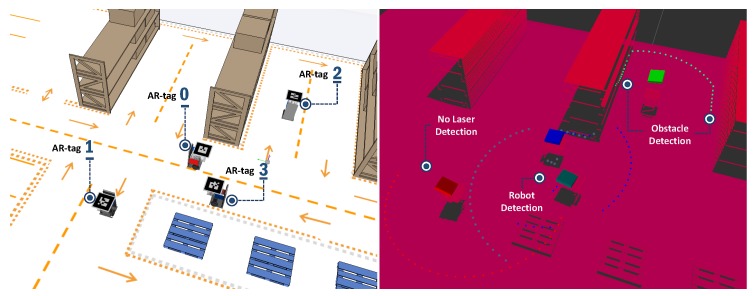
Example of virtual LRFs in the ARENA.

Data published by virtual LRF sensors can be used to map the environment and avoid obstacles. Figure 10 presents the ARENA with virtual sensors, identifying each robot according to their respective AR-tag. The virtual LRFs have a 5° resolution between each laser beam, totaling 37 equally spaced beams, resulting in a range of 0–180° measurement angle. Three distinct situations are found in Figure 10. The first refers to Robot 1, which has no detectable obstacles in the sensor range, although the pallets being close are undetectable as they are below the sensor. The second situation refers to Robot 2, which the virtual sensor detects both left and right racks, as it is within the sensor range. Finally, the third situation refers to the mutual perception between Robots 0 and 3. The virtual sensor of these two robots, besides detecting the rack, also detects the presence of the other robot.

## 4. Experimentation in the ARENA

**Figure 11:**
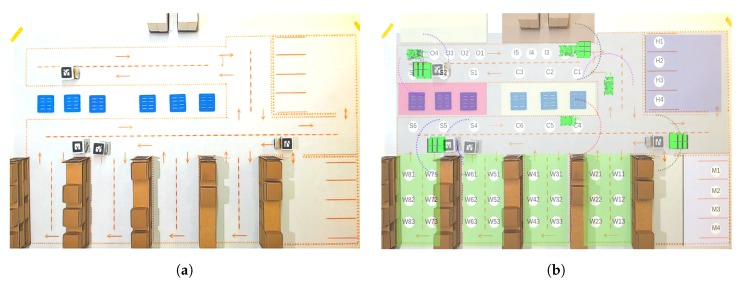
Improvements of AR in the ARENA: (**a**) top view of ARENA only with real elements; and (**b**) top view of ARENA with AR.

The ARENA employs the AR to promote realistic and highly immersive experimentation, allowing the visual understanding of warehouse logistics, production zones, and logistic states. The virtual elements improve the experimentation, not only in a more intuitive view, but enabling a more extensive analysis of smart behaviors, where the experimentation is shown in a raw view (Figure 11a) and with AR (Figure 11b).

**Figure 12:**
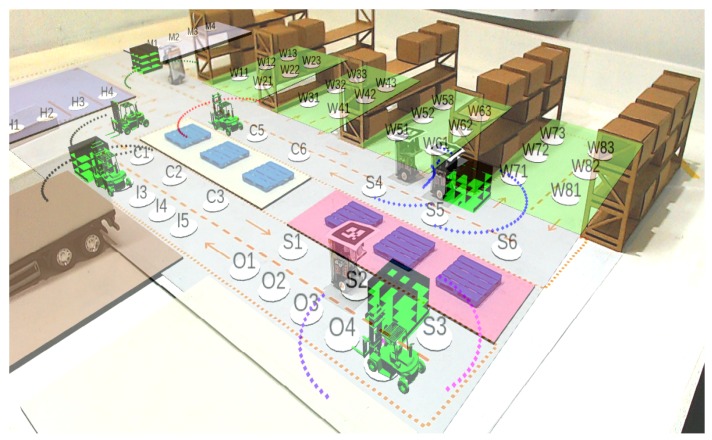
ARENA in perspective with AR.

An example of complete experimentation in the ARENA is illustrated in Figure 12, containing WsBots (as real forklifts) and virtual forklifts, both with LRF. These agents are handling pallet boxes, through the sequence specified in the warehouse logistics state machine (Figure 3). The virtual perception allows the tiny robot to perceive the industrial environment, understanding obstacles, and allowing intelligent and autonomous behaviors.

**Figure 13:**
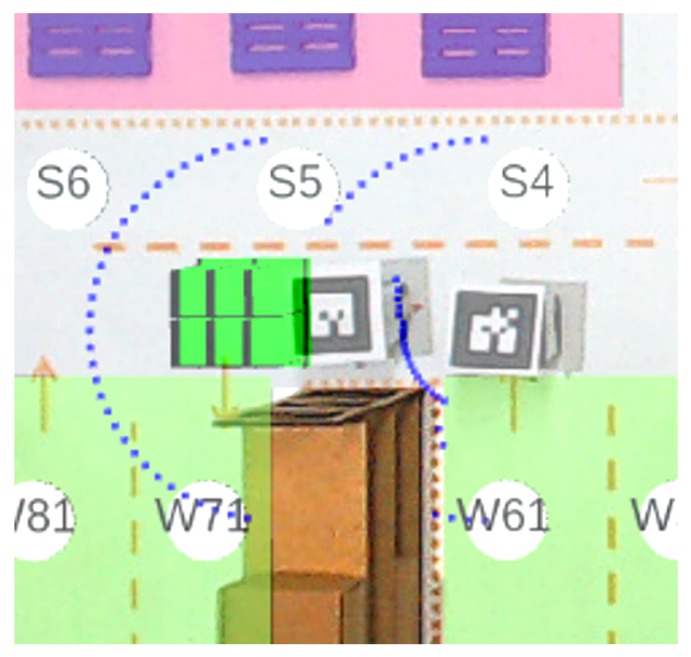
Example of obstacle detection with virtual LRF.

WsBots are tiny robots specially designed for experimentation in the small-scale warehouse, with the reduced payload. Thus, the use of virtual LRF is a unique solution to introduce autonomous behaviors as obstacle avoidance, environment mapping and cooperative handling. Figure 13 exemplifies the LRF sensor, where is detected the warehouse racks and a WsBot in front of observation robot.

### Accuracy and Precision of Virtual Sensor

A virtual scenario is developed to quantify the accuracy of virtual laser range finders. This setup consists of a cylindrical wall with no surface at the base for the location system to detect the robot’s AR-tag by providing the position of the virtual sensor. WsBot is centered on the cylinder axis so that all laser beams detect approximately the same distance from the wall. As ground truth of the setup in the simulator was placed, with the same orientation, on the AR-tag of WsBot, a 2D LRF sensor widely adopted by the robotics community, the Hokuyo URG-04LX. Both sensors were configured with the same parameters, thus a scan with a range from 0° to 180°, with a step angle of 5° resulting in 37 measurements per scan, and a maximum measuring distance of 1 m.

In the validation, five experiments were performed with the setup described above, where in the first test the cylinder radius was set to 0.15 m, and each new analysis was increased 0.05 m in this radius. For each experiment, ten ground truth and virtual LRF sensor scan samples were acquired. Table 2 presents the maximum, minimum, and average data of these ten samples. The difference between the Hokuyo beams and the same virtual sensor beams was analyzed, and the maximum differences, minimum differences, average differences, and, finally, standard deviation were measured.

The data shown in Table 2 are the comparison of maximum and minimum limits between the Hokuyo sensor and the virtual sensor, where it is noted that the virtual sensor has a more extensive range, resulting from the variation of the AR-tag location system. When analyzing the errors between the laser beams, it is noted that as the distance increases from the obstacle, the error increases, but does not compromise the accuracy of the virtual sensor.

## 5. Conclusions

This paper presents the ARENA, a platform that represents small-scale warehouse logistics, inspired by a real warehouse. The objective is to perform experimentation’s inside Industry 4.0, achieving intelligent behaviors aiming to improve manufacturing productivity. The ARENA provides an experience that benefits the development of autonomous and robust systems dedicated to the smart warehouse, using augmented reality-based techniques.

This work also discusses an MRS specially developed to operate in this ARENA, as autonomous forklifts, and the AR that expands the immersive experience and represents logistic stages. The agents can achieve autonomous behaviors with the use of virtual LRF, adding the capability of environment perception and obstacle avoidance. Several virtual elements are introduced to improve intelligent manufacturing experimentation without the use of a real warehouse. This strategy reduces the time and operational costs of developing new approaches by creating an ecosystem for experiments to increase operational successes.

Future works intend to perform task allocation experiments on a fault-tolerant system with multiple WsBot. A case study is also planned to evaluate AGV control algorithms, warehouse control, and management, and task scheduling to optimize production systems. For this, the ARENA will be applied to realistic and immersive experimentation.

## Figures and Tables

**Table 1:** Mechanical characteristics of WsBot.

Characteristic	Dimension	Unit
width	33.0	mm
length	33.0	mm
height	70.0	mm
wheel diameter	8.0	mm
wheel thickness	2.5	mm
mass	75.0	g

**Table 2:** Numerical comparison between Hokuyo LRF and proposed virtual LRF.

Cylinder Wall	Hokuyo (m)			Virtual LRF (m)			Error (m)			
Radius (m)	Max	Min	Avg	Max	Min	Avg	Max	Min	Avg	Std
0.15	0.1556	0.1456	0.1499	0.1514	0.1404	0.1451	0.0065	0.0031	0.0049	0.0007
0.20	0.2075	0.1963	0.2012	0.2031	0.1903	0.1958	0.0076	0.0033	0.0053	0.0010
0.25	0.2597	0.2481	0.2532	0.2558	0.2432	0.2487	0.0064	0.0001	0.0045	0.0009
0.30	0.3028	0.2900	0.2957	0.3003	0.2867	0.2919	0.0065	0.0013	0.0038	0.0013
0.35	0.3525	0.3401	0.3457	0.3502	0.3350	0.3416	0.0070	0.0010	0.0041	0.0015
